# Anlotinib exerts anti‐cancer efficiency on lung cancer stem cells in vitro and in vivo through reducing NF‐κB activity

**DOI:** 10.1111/jcmm.16564

**Published:** 2021-05-06

**Authors:** Zhuohong Li, Juncai Tian, Lei Du, Ying Gao, Yao Wang, Fengming You, Li Wang

**Affiliations:** ^1^ Department of Oncology Hospital of Chengdu University of Traditional Chinese Medicine Chengdu China; ^2^ Lung Cancer Center State Key Laboratory of Biotherapy West China Hospital of Sichuan University Chengdu China; ^3^ Department of Respiratory Medicine The First People’s Hospital of Ziyang Ziyang China

**Keywords:** anlotinib, apoptosis, cancer stem cell, NF‐κB

## Abstract

Anlotinib is a multi‐target tyrosine kinase inhibitor. Previous studies confirmed that anlotinib exerts anti‐cancer efficiency. However, the functional roles of anlotinib on cancer stem cells (CSCs) are yet to be elucidated. In this study, lung CSCs were isolated and identified in vitro, and mouse xenografts were established in vivo. MTT assays, tumour sphere formation assays, TdT‐mediated dUTP nick‐end labelling (TUNEL) staining, Annexin V‐FITC/PI staining, immunofluorescence analysis and Western blot were performed to investigate the anti‐cancer effects of anlotinib on lung CSCs. The results showed that anlotinib inhibits the growth of lung CSCs in vitro and in vivo. In addition, anlotinib induced apoptosis of these cells along with down‐regulated expression level of Bcl‐2 whereas up‐regulated Bax and cleaved caspase‐3 expression. It also sensitized lung CSCs to the cytotoxicity of cisplatin and paclitaxel; the tumour sphere formation and expression levels of multiple stemness‐associated markers, such as ALDH1 and CD133, were also decreased. Furthermore, the underlying mechanism indicated that anlotinib reduces the phosphorylated levels of NF‐κB p65 and IκB‐α in lung CSCs. Taken together, these findings suggested that anlotinib exerts potent anti‐cancer effects against lung CSCs through apoptotic induction and stemness phenotypic attenuation. The mechanism could be associated with the suppression of NF‐κB activity.

## 1 INTRODUCTION

Lung cancer is the most commonly diagnosed cancer and the first leading cause of cancer‐related deaths worldwide, according to recent cancer statistics in 2018.^1^ Despite benefits from surgical resection, systemic chemotherapy, radiotherapy and targeted therapy applied individually or in combination, therapeutic limitations are evident and developing new agents or therapeutic strategies for the management of lung cancer patients, especially advanced patients, are an urgent requirement.^2^, ^3^

Recently, accumulating evidence suggested that cancer stem cells (CSCs), a small heterogeneous cell population within tumour tissues or cancer cell lines, endowed with stemness phenotypic features including enhanced self‐renewal ability in vitro and in vivo and resistance to chemotherapy and high expression levels of stemness‐associated markers, such as ALDH1, CD133, Sox2 and Oct4, might be responsible for tumour recurrence and metastasis in cancer patients irrespective of comprehensive anti‐cancer therapies, including chemoradiotherapy, targeted therapy and immunotherapy.^4^, ^5^, ^6^ In the lung cancer cell lines or lung cancer patients, CSCs were isolated and involved in drug resistance and tumour recurrence.^7^, ^8^ Therefore, developing new anti‐cancer agents targeting lung CSCs (LCSCs) and exploring the molecular mechanisms underlying these processes are promising.

Anlotinib is a tyrosine kinase inhibitor targeting VEGFR, PDGFR, FGFR and c‐Kit.^9^ Previous studies reported that anlotinib exerts potent anti‐cancer efficiency against several types of cancer, including thyroid cancer, osteosarcoma and renal cell carcinoma.^10^, ^11^, ^12^ Recently, Hu *et al* clarified that anlotinib inhibits proliferation and induces apoptosis of KRAS‐mutated lung cancer cells.^13^ However, the anti‐cancer activities of anlotinib against LCSCs and the underlying molecular mechanism have not yet been elucidated.

In this study, we aimed to assess the potential functional roles of anlotinib on LCSCs in vitro and in vivo and explore the molecular mechanism involved in this process.

## 2 MATERIALS AND METHODS

### 2.1 Cell lines and culture

The human lung adenocarcinoma cell lines, PC‐9 and HCC827, purchased from Shanghai Cell Biology Institute of Chinese Academy of Sciences (Shanghai, China), were cultured in RPMI 1640 containing 10% FBS, penicillin (100 U/mL) and streptomycin (100 μg/mL) in a humidified environment with 5% CO_2_ at 37°C.

The PC‐9‐ and HCC827‐derived LCSCs were enriched using a non‐adhesive culture system, as described previously.^14^ Briefly, 1 × 10^6^ PC‐9 cells or HCC827 cells were seeded in culture plates pre‐coated with 0.5% agarose, respectively, and cultured with RPMI 1640 containing 10% FBS, penicillin (100 U/mL) and streptomycin (100 μg/mL) in a humidified environment with 5% CO_2_ at 37°C. The medium was changed every other day until tumour sphere formation within 5‐7 days. Passage 9 of PC‐9‐ and HCC827‐derived LCSCs were used to identify the stemness phenotype, including drug resistance, tumour sphere formation and the expression levels of stemness‐associated markers. Subsequently, passage 10 of PC‐9‐ and HCC827‐derived LCSCs were used in the subsequent experiments.

### 2.2 MTT assay

The cytotoxic effects of anlotinib (a gift from Zhengda Tianqing Pharmaceutical Group Co. Ltd), cisplatin (Sigma‐Aldrich) and paclitaxel (Sigma‐Aldrich) were analysed by MTT assay. Briefly, PC‐9 cells, HCC827 cells, PC‐9‐derived LCSCs or HCC827‐derived LCSCs (2 × 10^3^/well) were seeded in 96‐well plates (Costar Corning) and incubated overnight, followed by supplementation with anlotinib (2.5, 5, 10, 20, 30, 40 or 50 μM), cisplatin (1, 2, 4, 8, 16, 32 or 64 μM) and paclitaxel (2, 4, 6, 8, 16, 32 or 64 nM) for 3 days, respectively. After treatment, 10 μL of 5 mg/mL MTT (Sigma‐Aldrich) was added to each well and incubated for an additional 4 h. Then, 100 μL of 10% SDS/0.01N HCL was added to each well and incubated at 37°C overnight to dissolve the formazan. The absorbance was measured at 570 nm on a microplate reader (Bio‐Rad). The cell viability (%) of the treatment group was determined against that of control cells.

### 2.3 Annexin V‐FITC/PI double staining

Apoptosis was assessed using an Annexin V‐FITC/PI apoptotic detection kit (Keygen, Biotech), according to the manufacturer's instructions. Briefly, PC‐9 cells, HCC827 cells, PC‐9‐derived LCSCs or HCC827‐derived LCSCs, administered anlotinib (5, 10 or 20 μM) for 3 days, were incubated with FITC‐conjugated Annexin V and PI at room temperature for 15 minutes away from light. Then, these cells were subjected to flow cytometry (BD FACSCalibur; BD Bioscience) and sorted into intact (Annexin V and PI double‐negative), early apoptotic (Annexin V‐positive), late apoptotic (Annexin V and PI double‐positive) and necrotic (PI‐positive) cells.

### 2.4 Tumour sphere formation assay

Single‐cell suspensions from PC‐9 cells, HCC827 cells, PC‐9‐derived LCSCs or HCC827‐derived LCSCs were plated on 96‐well plates at a density of 50 cells/well, respectively. After 12 days of culture with or without anlotinib (10 or 20 μM), the spheres (50‐100 μm/sphere) in each well were counted under a light microscope. The sphere formation efficiency (SFE) was calculated as the number of spheres formed divided by the initial number of single cells plated and was expressed as a percentage.

### 2.5 Animals and ethics statement

All studies were approved by the Institutional Animal Care and Use Committee of Sichuan University (SCU). All animal feeding and experiments were carried out in accordance with the guidelines of the Institutional Animal Care and Use Committee at SCU.

Five‐week‐old female nude mice were purchased from the Experimental Animal Center, Sichuan University. Each animal was inoculated with 1 × 10^6^ PC‐9‐derived LCSCs subcutaneously in the right flank. After allowed to grow for 1 week to reach a tumour volume of approximately 50‐100 mm^3^, the animals were randomly divided into three groups (n = 6). The treatment group animals were treated with anlotinib at the concentrations of 10 and 20 mg/kg, and the control group animals were treated with normal saline intraperitoneally once daily for 4 weeks. Throughout the experiment, the tumour volumes were assessed by measuring the respective lengths and widths with vernier callipers. The tumour volumes were derived using the standard formula: V = width^2^ × length × 0.5. At the end of the treatment, the animals were euthanized by carbon dioxide asphyxiation, and tumour tissues were extracted, weighed and processed for fluorescence staining and Western blot analysis.

### 2.6 TUNEL staining

The apoptosis detection in tumour tissues was carried out through a One Step TUNEL Apoptosis Assay Kit (Beyotime Institute of Biotechnology), according to the manufacturer's instructions. Briefly, frozen section (5 μm) of tumour tissues from animal experiments mentioned above were plated onto Matrigel‐coated glass coverslips and permeabilized with 0.2% Triton X‐100 for 10 min at room temperature. Then, the sections were labelled using the TdT reaction and incubated for 1 h at 37°C. The DAPI (Sigma‐Aldrich) was used for nuclear staining. Finally, the apoptotic cells were visualized and counted under a fluorescence microscope (Leica).

### 2.7 Immunofluorescence staining

PC‐9‐ or HCC827‐derived LCSCs were permeabilized in 0.2% Triton X‐100 for 10 minutes at room temperature and then blocked with 5% BSA for 30 minutes. Then, the cells were incubated with primary antibody NF‐κB p65 (1:500; Cell Signaling Technology (CST)) at 4℃ overnight, followed by secondary antibody (ZSGB‐BIO) for 1 hour at 37°C for immunofluorescence staining. Finally, the cell nuclei were stained by DAPI for 10 minutes, and images were captured under a fluorescence microscope.

Frozen sections (5 μm) of tumour tissues from animal experiments mentioned above were assessed for ALDH1 and CD133 expression. The sections were fixed in 4% paraformaldehyde, blocked with 5% BSA for 30 minutes and incubated at 4°C overnight with the following primary antibodies: ALDH1 and CD133 (1:1000, Abcam). Then, the cells were incubated with appropriate secondary antibodies for 1 hour at 37°C. DAPI was used to visualize nuclei. The images were captured under a fluorescence microscope linked to a camera, and the fluorescence intensities were measured using the Image J software (NIH).

### 2.8 Western blot analysis

Cells or tumour tissues from mice were treated with a Nuclear and Cytoplasmic Protein Extraction Kit or RIPA lysis buffer supplemented with protease inhibitor cocktail and protein phosphatase inhibitor (Beyotime Biotechnology). An equivalent of 20 µg was separated by 8‐12% SDS‐PAGE and transferred to polyvinylidene fluoride (PVDF) membranes (Millipore) by electroblotting. The membranes were probed with specific antibodies against Bcl‐2, Bax, cleaved caspase‐3, pho‐NF‐κB p65, pho‐IκB‐α, IκB‐α and NF‐κB p65 (1:1000; CST), β‐actin, GAPDH and Histone H3 (1:1000; Beijing Biosynthesis Biotechnology Co., Ltd), and ALDH1, CD133, Oct4 and Sox2 (1:1000; Abcam). Subsequently, the membranes were incubated with horseradish peroxidase (HRP)‐conjugated secondary antibodies. Antibody‐bound proteins were detected by BeyoECL Plus kit (Beyotime Institute of Biotechnology) and Western blot analysis (Universal Hood II, Bio‐Rad).

### 2.9 Statistical analysis

Data were analysed by GraphPad Version 6.0 and expressed as mean ± standard deviation (SD). Significant differences between groups were analysed using Student's *t* test and one‐ or two‐way analysis of variance (ANOVA). Statistical significance was defined as *P* < 0.05 for all tests.

## 3 RESULTS

### 3.1 Identification of stemness phenotypic characteristics in PC‐9‐ and HCC827‐derived LCSCs

**FIGURE 1:**
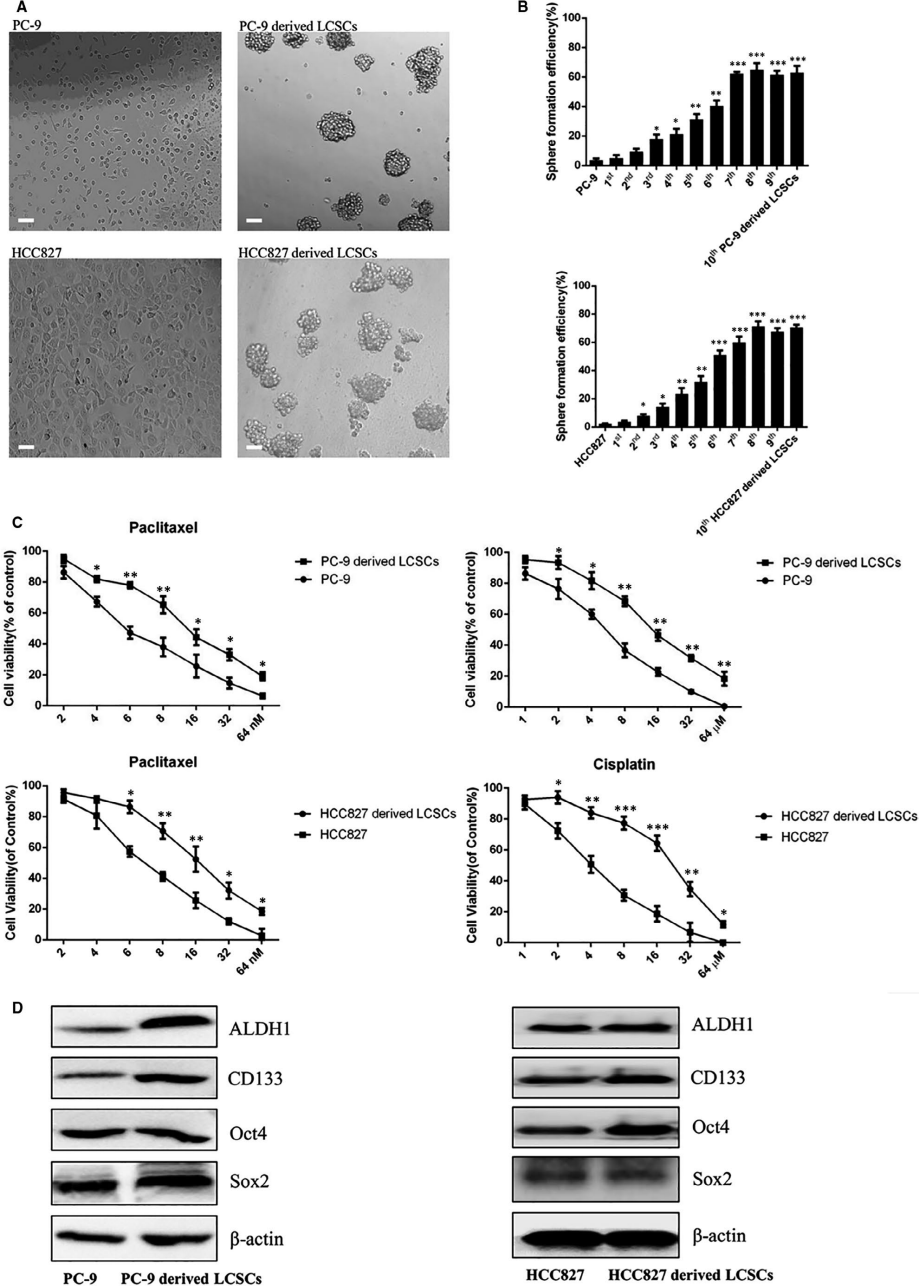
Identification of stemness phenotypic characteristics of PC‐9‐ and HCC827‐derived LCSCs. A, The morphological observation of PC‐9 cells, PC‐9‐derived LCSCs, HCC827 cells and HCC827‐derived LCSCs; scale bar = 100 μm. B, Cell viability of PC‐9 cells, PC‐9‐derived LCSCs, HCC827 cells and HCC827‐derived LCSCs after treatment with various concentrations of paclitaxel and cisplatin, respectively. PC‐9 vs. PC‐9‐derived LCSCs and HCC827 vs. HCC827‐derived LCSCs. C, The histograms show tumour sphere formation efficiency of PC‐9 cells, PC‐9‐derived LCSCs, HCC827 cells and HCC827‐derived LCSCs, respectively. PC‐9 vs. 1st, 2nd, 3rd……10th PC‐9‐derived LCSCs; HCC827 vs. 1st, 2nd, 3rd……10th HCC827‐derived LCSCs. D, Western blot analysis of ALDH1, CD133, Sox2 and Oct4 among PC‐9 cells, PC‐9‐derived LCSCs, HCC827 cells and HCC827‐derived LCSCs. PC‐9 vs. PC‐9‐derived LCSCs; HCC827 vs. HCC827‐derived LCSCs. * *P* < 0.05; ** *P* < .01; *** *P* < 0.001. Data are expressed as mean ± SD of three independent experiments performed in triplicate

As shown in Figure 1A, PC‐9‐ and HCC827‐derived LCSCs were grown as suspension cultures and exhibited a sphere‐like morphology, which was remarkably different from parental PC‐9 cells and HCC827 cells, respectively. In addition, the sphere formation assay revealed that along with increasing passages of PC‐9‐ and HCC827‐derived LCSCs, the SFE of these cells was enhanced gradually compared with the SFE in parental PC‐9 cells and HCC827 cells, respectively (Figure 1B). Moreover, the PC‐9‐ and HCC827‐derived LCSCs were more resistant to cisplatin and paclitaxel cytotoxicity than PC‐9 cells and HCC827 cells, respectively. The inhibitory concentration 50 (IC_50_) values of PC‐9‐derived LCSCs were 15.91 ± 0.34 µM (cisplatin) and 16.45 ± 0.57 nM (paclitaxel), respectively; both values were much higher than that obtained for PC‐9 cells (4.16 ± 0.81 µM and 6.86 ± 0.72 nM). For HCC827‐derived LCSCs, the IC_50_ values were 17.32 ± 0.41 µM (cisplatin) and 18.28 ± 0.63 nM (paclitaxel), respectively, and both were much higher than that obtained for HCC827 cells (3.96 ± 0.72 µM and 7.73 ± 0.32 nM) (Figure 1C). Western blot analysis demonstrated that ALDH1, CD133, Sox2 and Oct4 were overexpressed in PC‐9‐derived LCSCs compared with PC‐9 cells; however, in HCC827‐derived LCSCs, ALDH1, CD133 and Oct4 were overexpressed compared with HCC827 cells (Figure 1D).

### 3.2 Anlotinib inhibits proliferation and induces apoptosis of PC‐9‐ and HCC827‐derived LCSCs

**FIGURE 2:**
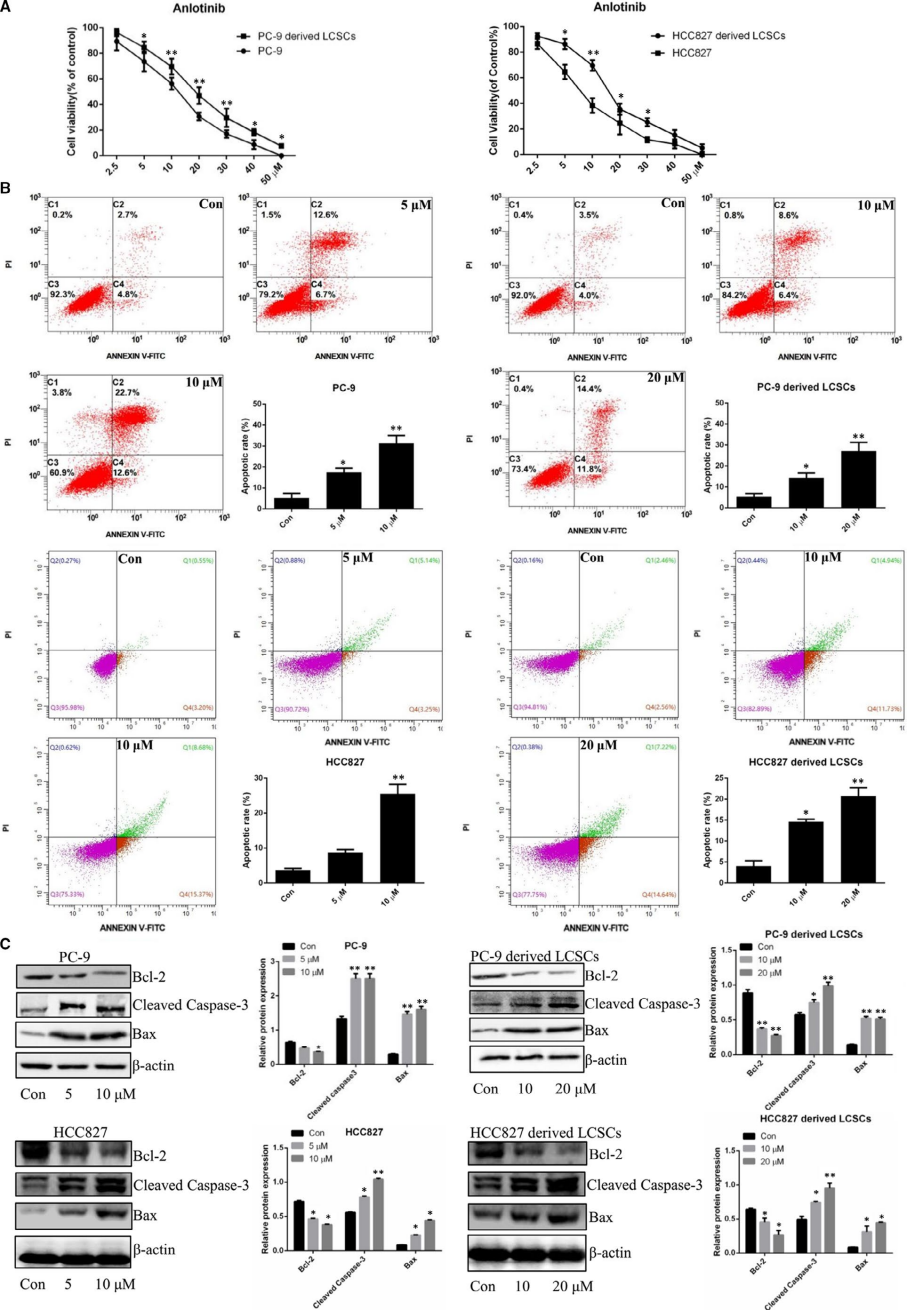
Anlotinib inhibits proliferation and induces apoptosis of PC‐9‐ and HCC827‐derived LCSCs. A, Cell viability of PC‐9 cells, PC‐9‐derived LCSCs, HCC827 cells and HCC827‐derived LCSCs after treatment with various concentrations of anlotinib. PC‐9 vs. PC‐9‐derived LCSCs; HCC827 vs. HCC827‐derived LCSCs. B, Annexin V‐FITC/PI double staining was used for apoptosis detection in PC‐9 vs. PC‐9‐derived LCSCs; HCC827 vs. HCC827‐derived LCSCs after treatment with various concentrations of anlotinib. Early and late apoptotic cells were assessed on flow cytometer; the histograms represent the proportions of early, late and total apoptotic cells, respectively. C, Western blot analysis of Bcl‐2, Bax and cleaved caspase‐3 in PC‐9 cells, PC‐9‐derived LCSCs, HCC827 cells and HCC827‐derived LCSCs after treatment with various concentrations of anlotinib. Con vs. 5, 10, or 20 μM. * *P* < 0.05; ** *P* < 0.01. Data are expressed as mean ± SD of three independent experiments performed in triplicate

The cytotoxicity of anlotinib on PC‐9 cells, HCC827 cells, PC‐9‐derived LCSCs and HCC827‐derived LCSCs was analysed through MTT assay. As shown in Figure 2A, anlotinib inhibited the proliferation of PC‐9 cells and PC‐9‐derived LCSCs with the IC_50_ values of 8.06 ± 1.2 μM and 15.73 ± 0.48 μM, respectively. In HCC827 cells and HCC827‐derived LCSCs, the effects of proliferation inhibition of anlotinib were also observed with the IC_50_ values of 7.39 ± 0.81 μM and 13.19 ± 0.53 μM, respectively. In order to determine whether anlotinib induced the loss of proliferation capacity of these cells above was associated with apoptosis, these cells were treated with anlotinib, and the proportion of apoptotic cells was analysed. As shown in Figure 2B, flow cytometry evaluated both early and late apoptotic PC‐9 cells and PC‐9‐derived LCSCs that were increased after treatment with different concentrations of anlotinib. For HCC827 cells and HCC827‐derived LCSCs, the proportion of apoptotic cells ascended in a dose‐dependent manner. Western blot analysis further verified that after treatment with anlotinib, the expression level of Bcl‐2 was down‐regulated, whereas the levels of Bax and cleaved caspase‐3 were up‐regulated in PC‐9 cells and PC‐9‐derived LCSCs, respectively. These results were also observed in anlotinib‐treated HC8227 cells and HCC827‐derived LCSCs (Figure 2C).

### 3.3 Anlotinib attenuates stemness phenotypic features of PC‐9‐ and HCC827‐ derived LCSCs

**FIGURE 3:**
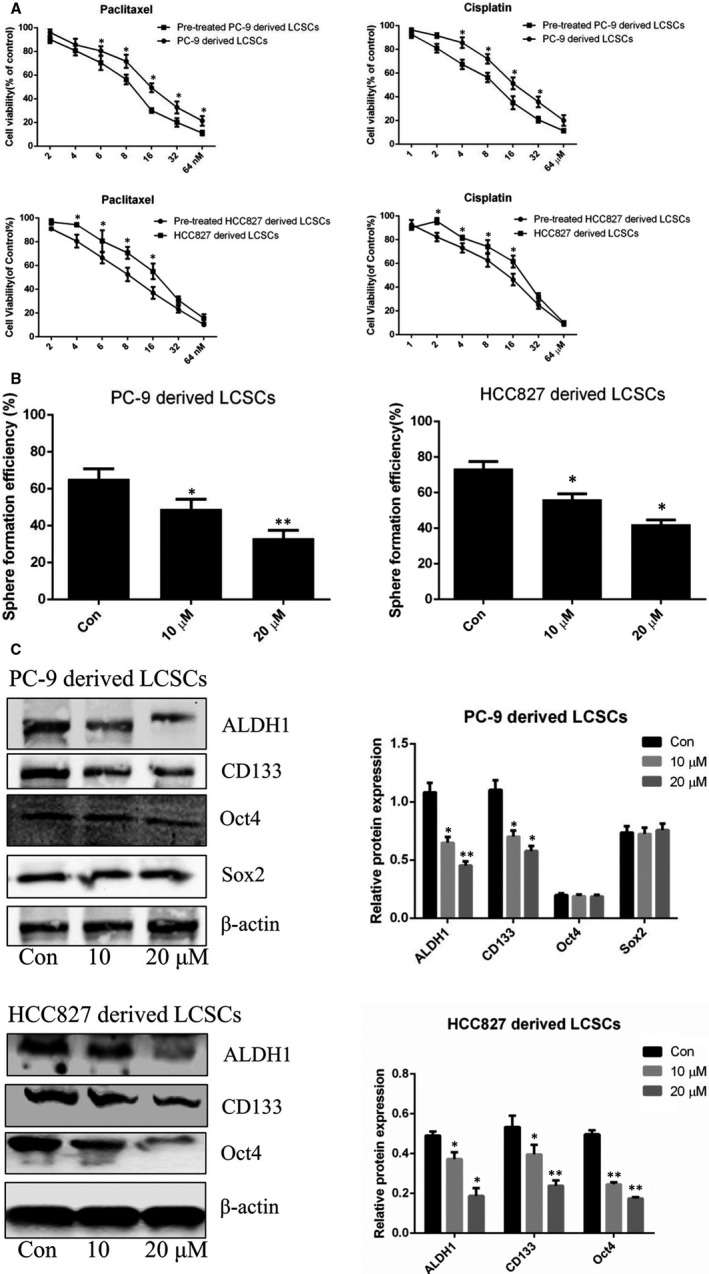
Anlotinib attenuates stemness phenotype of PC‐9‐ and HCC827‐derived LCSCs. A, Cell viability of anlotinib (20 μM) pre‐treated PC‐9‐ and HCC827‐derived LCSCs after administration of various concentrations of paclitaxel and cisplatin, respectively. Pre‐treated PC‐9‐derived LCSCs vs. PC‐9‐derived LCSCs; Pre‐treated HCC827‐derived LCSCs vs. HCC827‐derived LCSCs. B, Histograms show tumour sphere formation efficiency of PC‐9‐ and HCC827‐derived LCSCs after treatment with various concentrations of anlotinib. C, Western blot analysis of ALDH1, CD133, Oct4 and Sox2 in PC‐9‐ and HCC827‐derived LCSCs after treatment with various concentrations of anlotinib. Con vs. 10 or 20 μM. * *P* < 0.05; ** *P <* 0.01. Data are expressed as mean ± SD of three independent experiments performed in triplicate

To investigate the effects of anlotinib on the stemness phenotype in PC‐9‐ and HCC827‐derived LCSCs, we assessed the drug resistance, SFE and the changes in the expression levels of stemness‐associated markers in these cells. As shown in Figure 3A, anlotinib sensitized PC‐9‐derived LCSCs to the cytotoxicity of cisplatin and paclitaxel, compared with the IC_50_ values (9.05 ± 1.17 µM and 10.81 ± 0.37 nM, respectively) of control cells not pre‐treated with anlotinib. For HCC827‐derived LCSCs pre‐treated with anlotinib, the IC_50_ values were 9.61 ± 0.33 µM and 10.15 ± 0.59 nM for the cytotoxicity of cisplatin and paclitaxel, respectively, which decreased compared with those obtained with control cells. Moreover, sphere formation assay revealed that anlotinib reduced the SFE of PC‐9‐ and HCC827‐derived LCSCs in a dose‐dependent manner (Figure 3B). Western blot analysis demonstrated that the expression levels of ALDH1, Sox2, and CD133 were down‐regulated in PC‐9‐derived LCSCs after anlotinib treatment. However, the levels of ALDH1, Oct4 and CD133 were decreased in anlotinib‐treated HCC827‐derived LCSCs (Figure 3C).

### 3.4 Anlotinib exerts anti‐cancer effects in vivo

**FIGURE 4:**
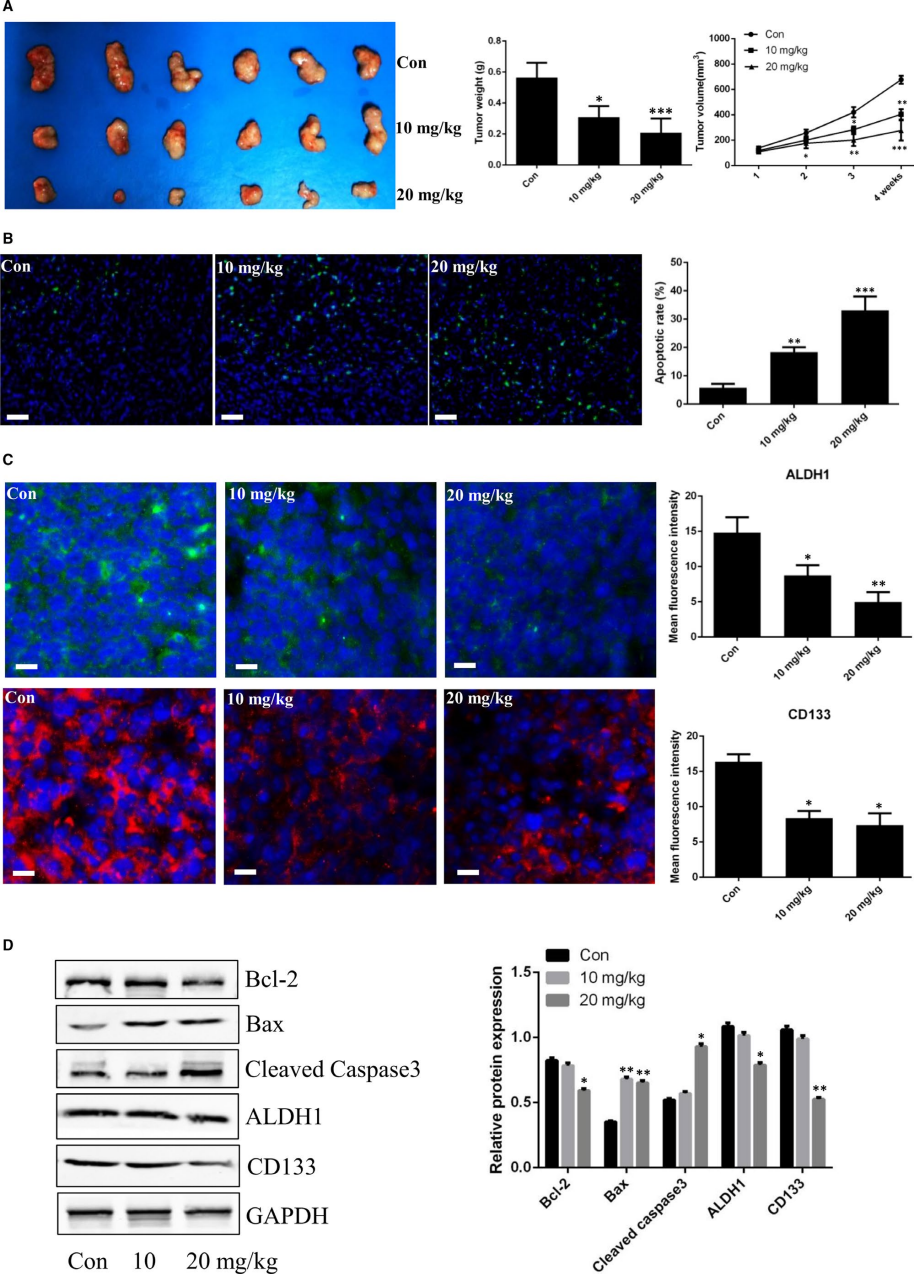
Anti‐cancer effects of anlotinib in PC‐9‐derived LCSC tumour‐bearing mice. A, Tumour volumes and weights were measured after treatment with anlotinib. B, TNUEL staining of xenografts after treatment with anlotinib. C, Immunofluorescence staining of CD133 and ALDH1 in xenografts after treatment with anlotinib; histograms show fluorescence intensity of CD133 and ALDH1. D, Western blot analysis of Bcl‐2, Bax, cleaved caspase‐3, CD133 and ALDH1 in xenografts after treatment with anlotinib. Con vs. 10 or 20 mg/kg. * *P* < 0.05; ** *P* < 0.01; *** *P* < 0.001. Data are expressed as mean ± SD of three independent experiments performed in triplicate

As demonstrated above, anlotinib induced apoptosis and attenuated the stemness phenotype of PC‐9‐ and HCC827‐derived LCSCs in vitro. However, whether anlotinib exerted anti‐cancer properties against LCSCs in vivo is yet unclear. To further assess the functional roles of anlotinib, PC‐9‐derived LCSC tumour‐bearing mice were established and intraperitoneally administered 10 mg/kg and 20 mg/kg anlotinib, respectively, for 4 weeks. As shown in Figure 4A, anlotinib significantly inhibited the tumour growth of PC‐9‐derived LCSC tumour‐bearing mice. In addition, TUNEL staining in xenografts demonstrated that anlotinib promoted apoptosis of PC‐9‐derived LCSCs in vivo along with decreased Bcl‐2 expression and increased levels of Bax and cleaved caspase‐3 (Figure 4B,D). Furthermore, immunofluorescence staining of ALDH1 and CD133 in xenografts verified that the fluorescence intensity of these stemness‐associated markers was decreased after administration of anlotinib in PC‐9‐derived LCSC tumour‐bearing mice. These findings were further verified by Western blot analysis in xenografts (Figure 4C,D).

### 3.5 Anlotinib suppresses NF‐κB activity in PC‐9‐ and HCC827‐derived LCSCs

**FIGURE 5:**
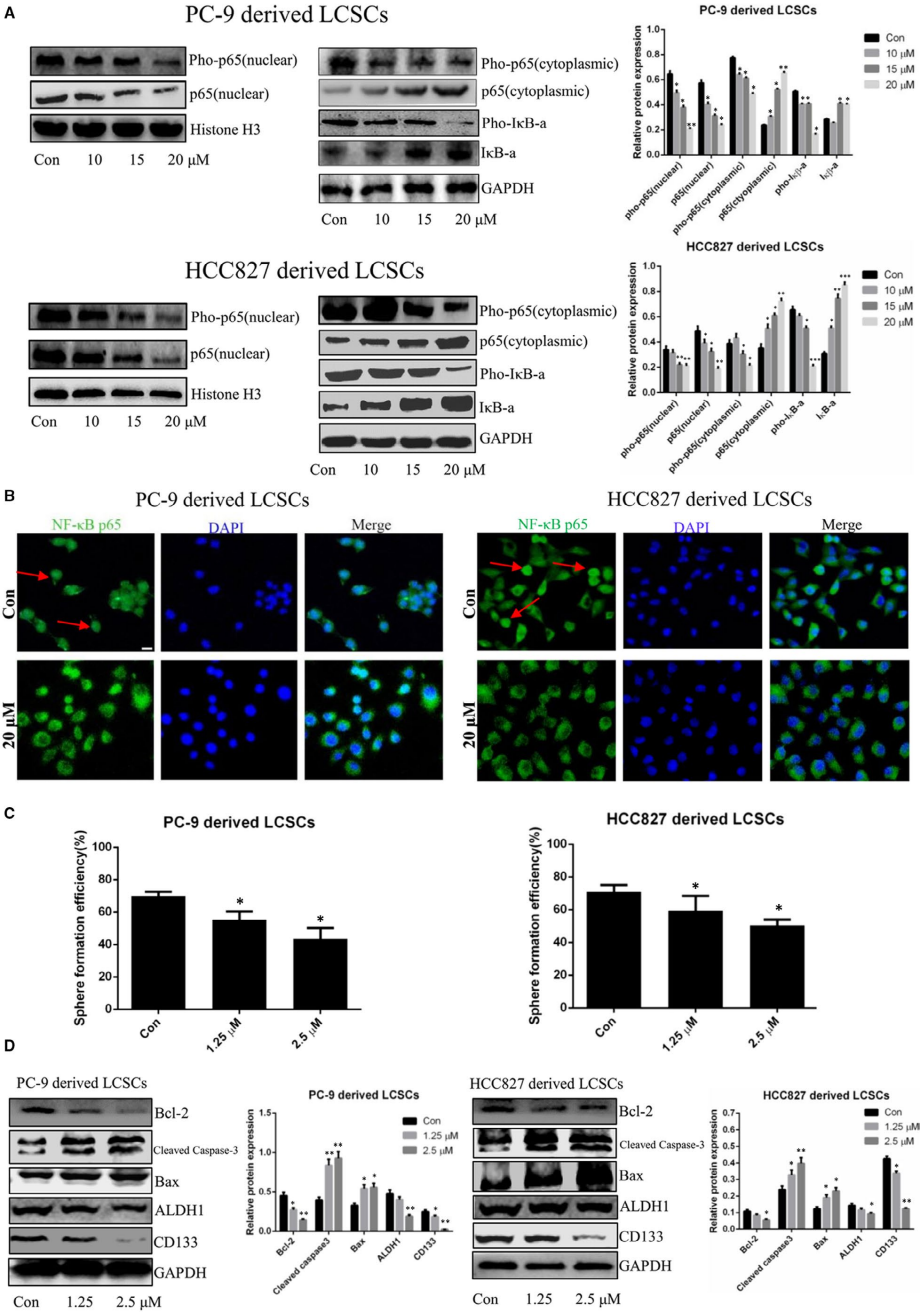
Anlotinib suppresses NF‐κB activity in PC‐9‐ and HCC827‐derived LCSCs. A, Western blot analysis of pho‐NF‐κB p65, NF‐κB p65, IκB‐α and pho‐IκB‐α in PC‐9‐ and HCC827‐derived LCSCs after treatment with various concentrations of anlotinib. Con vs. 10, 15, or 20 μM. * *P* < 0.05; ** *P* < 0.01; *** *P* < 0.001. Data are expressed as mean ± SD of three independent experiments performed in triplicate. B, Immunofluorescence staining of NF‐κB p65; the cells with nuclear translocation of NF‐κB p65 are indicated with red arrows; the scale bar = 100 μm. C, Histograms show tumour sphere formation efficiency of PC‐9‐ and HCC827‐derived LCSCs after treatment with various concentrations of BAY11‐7082. D, Western blot analysis of Bcl‐2, cleaved caspase‐3, Bax, ALDH1 and CD133 in PC‐9‐ and HCC827‐derived LCSCs after treatment with various concentrations of BAY11‐7082. Con vs. 1.25 or 2.5 μM. * *P* < 0.05; ** *P* < 0.01. Data are expressed as mean ± SD of three independent experiments performed in triplicate

Previous reports indicated that the regulation of NF‐κB pathway was closely associated with apoptosis and stemness phenotype maintained in CSCs.^15^, ^16^, ^17^, ^18^ To clarify the effects of anlotinib on apoptosis and stemness phenotype attenuation in PC‐9‐ and HCC827‐derived LCSCs involved with the regulation of NF‐κB activity, we examined the changes in the NF‐κB pathway protein expression post‐anlotinib treatment. As shown in Figure 5A, the expression level of cytoplasmic NF‐κB p65 was up‐regulated, whereas the nuclear level was down‐regulated. Strikingly, the phosphorylated levels of NF‐κB p65 in both cytoplasm and nuclei were reduced after anlotinib treatment. On the other hand, anlotinib treatment decreased the phosphorylated level of IκB‐α in PC‐9‐ and HCC827‐derived LCSCs. Fluorescence staining of NF‐κB p65 further verified that anlotinib blocked the translocation of NF‐κB p65 to the nucleus in PC‐9‐ and HCC827‐derived LCSCs (Figure 5B). Together, these results suggested that anlotinib suppressed NF‐κB activity in PC‐9‐ and HCC827‐derived LCSCs. In order to further explore whether the regulation of NF‐κB pathway is related to apoptosis induction and stemness phenotype attenuation in these cells, BAY11‐7082 (Beyotime Institute of Biotechnology), a specific NF‐κB inhibitor, was utilized. As shown in Figure 5C, sphere formation assay demonstrated that BAY11‐7082 decreased the SFE of PC‐9‐ and HCC827‐derived LCSCs. In addition, the expression level of stemness‐associated markers (ALDH1 and CD133) was reduced after BAY11‐7082 treatment (Figure 5D). We also observed that in BAY11‐7082 treated PC‐9‐ and HCC827‐derived LCSCs, the level of anti‐apoptotic Bcl‐2 protein was down‐regulated, whereas that of pro‐apoptotic proteins Bax and cleaved caspase‐3 was up‐regulated in a dose‐dependent manner (Figure 5D). These data indicated that anlotinib‐induced apoptosis and anlotinib ‐attenuated stemness phenotype of PC‐9‐ and HCC827‐derived LCSCs might at least partially, be attributed to the suppression of NF‐κB activity.

## 4 DISCUSSION

Lung cancer is one of the most lethal malignancies, and the 5‐year survival rate is <18%. Despite improvements in the present therapies of lung cancer, including the expansion of indications for surgical resection, modification of chemotherapeutic drugs, accurate radiotherapy, continuous development of targeted drugs and burgeoning immunotherapy, the anti‐cancer efficiency is still limited. Advanced lung cancer patients experience tumour recurrence and metastasis after a series of therapeutic methods against cancer. Therefore, exploring the pathogenesis of lung cancer and developing new targeted agents is an urgent requirement.

CSCs, also known as tumour‐initiating cells, constitute a small heterogeneous cell population within many types of cancers, including lung, breast, brain, pancreas and colon.^19^ Though the proportion of CSCs is low, approximately <6%‐7%, the cells are endowed with stemness phenotype characteristics, including enhanced self‐renewal ability, resistance to chemotherapy and high expression levels of stemness markers, such as ALDH1, Sox2, Oct4 and CD133.^20^ Current evidence suggests that the presence of CSCs drives the metastasis and recurrence of tumour; thus, eliminating CSCs might be a promising strategy combined with chemotherapy or other anti‐cancer therapies.^21^ Together, the establishment of the CSC model is imperative. In this study, we utilized the non‐adhesive culture system and isolated and identified the stemness phenotype of lung CSCs from parental PC‐9 cells and HCC827 cells.

CSCs are characterized by self‐renewal ability to maintain their proportion in tumours and the potential to differentiate into non‐tumorigenic bulk tumour cells; the self‐renewal is tightly associated with the pathological ability to regenerate tumours.^15^ The sphere formation assay is an in vitro method commonly used as a functional reporter of the self‐renewal ability of CSCs.^22^, ^23^ Yang *et al* reported that the SFE in primary and secondary A549‐dervied LCSCs was much higher than that in parental A549 cells.^24^ In addition, Li *et al* verified that cervical cancer cell‐derived CSCs exhibited increased SFE compared with parental cervical cancer cells, and the efficiency of sphere formation in these CSCs stabilized after a series of passages.^14^ In the present study, the sphere formation assay demonstrated that the SFE of PC‐9‐ and HCC827‐derived LCSCs was increased gradually and also tended to be stable after experiencing multiple and serial propagations of the spheres compared with the parental PC‐9 cells and HCC827 cells, respectively. Resistance to the cytotoxicity of chemotherapeutic agents is an important trait of CSCs. Kanwal *et al* demonstrated that CD133‐positive prostate cancer cells were endowed with stem cell‐like characteristics, including therapeutic resistance to docetaxel, which is a chemotherapeutic drug acting efficiently on CD133‐negative prostate cancer cells.^25^ In addition, He *et al* demonstrated that breast cancer cell line BT549‐derived CSCs were resistant to the cytotoxicity of doxorubicin, commonly used as a systemic therapeutic drug for breast cancer patients.^26^ In this study, we also verified that PC‐9‐ and HCC827‐derived LCSCs were resistant to cisplatin and paclitaxel, indicating that CSCs might be responsible for the drug resistance cancer patients despite multiline chemotherapy. The stemness‐associated markers were previously reported to maintain the phenotypic features in CSCs, and to decrease or to increase the expression levels of these markers could influence the self‐renewal ability, chemotherapy resistance or metastatic potential of these cells. Recently, Zhan *et al* reported that overexpressing Sox2 promoted the stemness phenotypic characteristics, such as colony formation and metastatic ability in bladder cancer cells.^27^ Li *et al* also indicated that silencing of ALDH1 expression through siRNA technology decreased the tumorigenicity and migration ability of breast CSCs.^28^ Moreover, Chen *et al* revealed that knockdown of Oct4 expression in lung CSCs significantly inhibited the abilities of tumour invasion and colony formation.^29^ CD133 is a common and vital stemness marker and identified in many types of CSCs, including lung, breast and prostate CSCs, as well as glioma stem cells.^30^, ^31^, ^32^ Ding *et al* verified that CD133 is a liver CSC marker, and reducing CD133 expression in these cells attenuated chemotherapeutic drug 5‐Fu resistance in hepatocellular carcinoma.^33^ In another study, Lan *et al* clarified that down‐regulated CD133 expression in CD133‐positive liver CSCs inhibits the tumour sphere formation, colony formation, and in vivo tumour growth while enhancing the sensitivity of liver CSCs to chemotherapy and radiotherapy.^34^ In the present study, we demonstrated that the expression level of stemness‐associated markers, including ALDH1, Sox2, Oct4 and CD133, was increased in PC‐9‐derived LCSCs compared with PC‐9 cells, which was consistent with increased stemness phenotype in these cells. However, in HCC827‐derived LCSCs, the expression level of Sox2 was low and similar to the parental HCC827 cells, suggesting that Sox2 might not be closely involved with the functional regulation of stemness phenotypic characteristics in HCC827 cells.

These results above indicated that our enriched PC‐9‐ and HCC827‐derived LCSCs were featured with stemness phenotype and could be used as CSC models for further investigation.

Anlotinib is a novel tyrosine kinase inhibitor (TKI) approved for advanced lung cancer patients due to obvious antiangiogenic effects in tumour tissues. Typically, anlotinib exerts anti‐cancer effects in several types of cancer cells. However, the effect of anlotinib on CSCs and the underlying molecular mechanisms remains unclear. In the present study, we first demonstrated that anlotinib inhibited the proliferation of PC‐9‐ and HCC827‐derived LCSCs in vitro.

Apoptosis is a critical mechanism of anti‐cancer drugs and is involved with cell proliferation inhibition. A previous study indicated that anlotinib induced apoptosis of KRAS mutant A549 cells. In this study, flow cytometry analysis clarified that anlotinib also induced apoptosis in PC‐9‐ and HCC827‐derived LCSCs. Apoptosis is essentially regulated by two large families of proteins: caspase and Bcl‐2.^35^ Bcl‐2 protects the cells from apoptosis, whereas Bax reverses the effect. The caspases are a family of protease enzymes that are mainly classified into initiators (caspase‐9) and effectors (caspase‐3). Based on these findings, we assessed the changes in the expression of apoptotic proteins in these cells after anlotinib treatment, and the results showed that the expression levels of Bax and cleaved caspase‐3 in PC‐9‐ and HCC827‐derived LCSCs were increased, whereas that of Bcl‐2 was decreased. In the in vivo assay, we established PC‐9‐derived LCSC tumour‐bearing mice and found that anlotinib suppressed tumour growth. Furthermore, TUNEL staining revealed that the proportion of TUNEL‐positive cells in xenografts of PC‐9‐derived LCSC tumour‐bearing mice was increased after anlotinib treatment in addition to the up‐regulated expression of Bax and cleaved caspase‐3 and the down‐regulated expression of Bcl‐2.

Next, we assessed the changes in stemness phenotypic characteristics in PC‐9‐ and HCC827‐derived LCSCs after anlotinib treatment. The results firstly verified that anlotinib decreased tumour sphere formation and chemotherapy resistance of these LCSCs in vitro. On the other hand, after treatment with anlotinib, the expression level of stemness‐associated markers, ALDH1 and CD133, was obviously reduced in PC‐9‐ and HCC827‐derived LCSCs as well as the xenografts of PC‐9‐derived LCSC tumour‐bearing mice.

Previous reports suggested that NF‐κB pathway was closely involved with apoptosis and the stemness phenotype of CSCs. Erdogan *et al* demonstrated that in prostate CSCs, apigenin induced extrinsic caspase‐dependent apoptosis in these cells by decreasing the expression level of NF‐κB p105 and NF‐κB p50.^15^ In leukaemia stem cells, Xu *et al* revealed that disulfiram combined with copper exerted pro‐apoptotic effects of these cells via suppression of NF‐κB p65 expression.^16^ Moreover, Zhang *et al* suggested that in pancreatic cancer, gemcitabine treatment activated NF‐κB signalling pathway by increasing the nuclear translocation of NF‐κB p65 and then promoted stemness phenotypic characteristics, such as enhanced sphere formation, migration and chemoresistance as well as the expression of stemness markers, including Sox2, Nanog and Bmi1.^17^ Similarly, ovarian CSCs presented high expression of NF‐κB pathway‐associated proteins, including RelA, RelB and IKKα. After inhibiting the NF‐κB pathway in these cells, the stemness phenotype was reduced, including the down‐regulated expression levels of stemness genes and decreasing clonogenic capacity and tumorigenesis.^18^ In the present study, we demonstrated that anlotinib suppressed NF‐κB activity through down‐regulating the phosphorylated levels of NF‐κB and IκB‐α in PC‐9‐ and HCC827‐derived LCSCs. In order to further clarify whether regulating NF‐κB activity was related to apoptosis induction and stemness phenotype attenuation in these cells, BAY11‐7082, a specific NF‐κB inhibitor, was employed. Previous studies suggested that BAY11‐7082 suppressed the NF‐κB activity in many types of cancers, including lung cancer, leukemic, prostate cancer and cervical cancer.^36^, ^37^, ^38^, ^39^ In this study, we demonstrated that BAY11‐7082 induces apoptosis and attenuates stemness phenotype in PC‐9‐ and HCC827‐derived LCSCs.

## 5 CONCLUSIONS

In summary, the current findings indicated that anlotinib exerts anti‐cancer efficiency on PC‐9‐ and HCC827‐derived LCSCs in vitro and in vivo via apoptosis induction and stemness phenotypic attenuation. This phenomenon is associated with the suppression of NF‐κB activity. Therefore, anlotinib may constitute a new agent for treating LCSCs, which needs to be investigated further.

## CONFLICT OF INTEREST

These authors declare that there is no conflict of interests regarding the publication of this article.

## AUTHOR CONTRIBUTIONS

**Li Wang:** Conceptualization (lead); Supervision (lead); Writing‐original draft (lead); Writing‐review & editing (lead). **Zhuohong Li:** Data curation (lead); Formal analysis (lead); Funding acquisition (supporting); Resources (lead); Software (lead). **Juncai Tian:** Conceptualization (equal); Data curation (lead); Formal analysis (lead); Resources (lead); Software (lead). **Lei Du:** Formal analysis (equal); Software (equal). **Ying Gao:** Data curation (equal); Software (equal). **Yao Wang:** Formal analysis (equal); Resources (equal); Software (equal). **Fengming You:** Methodology (lead); Supervision (lead); Writing‐original draft (lead); Writing‐review & editing (lead).

## Data Availability

The datasets used and/or analysed during the current study are available from the corresponding author upon reasonable request.
